# Fracture strength of endodontically treated lateral incisors restored with new zirconia reinforced rice husk nanohybrid composite

**DOI:** 10.4317/jced.56864

**Published:** 2020-08-01

**Authors:** Galvin-Sim-Siang Lin, Nik-Rozainah-Nik-Abdul Ghani, Noor-Huda Ismail, Kiran Singbal, Devarasa-Giriyapura Murugeshappa, Noraida Mamat

**Affiliations:** 1 Conservative Dentistry Unit, School of Dental Sciences, Universiti Sains Malaysia, Health campus, 16150, Kubang Kerian, Kota Bharu, Kelantan, Malaysia; 2 Prosthodontic Unit, School of Dental Sciences, Universiti Sains Malaysia, Health Campus, 16150, Kubang Kerian, Kota Bharu, Kelantan, Malaysia; 3 Department of Restorative Dentistry, Faculty of Dentistry, Mahsa University, Bandar Saujana Putra, 42610, Selangor, Malaysia; 4 Paediatric Dentistry Unit, School of Dental Sciences, Universiti Sains Malaysia, Health campus, 16150, Kubang Kerian, Kota Bharu, Kelantan, Malaysia

## Abstract

**Background:**

This study aimed to compare the fracture strength, fracture pattern and type of fracture of endodontically treated maxillary lateral incisors restored with new zirconia reinforced rice husk nanohybrid composite.

**Material and Methods:**

Eighty mature permanent maxillary lateral incisors from patients age range of 30-60 years with single canal were selected and randomly divided into: Group 1 – RCT + nanofilled composite (Filtek), Group 2 – RCT + microhybrid composite (Zmack), Group 3 – RCT + new nanohybrid composite (Zr-Hybrid) and Group 4 - Intact teeth (control). Standardized mesio-palatal-distal cavity was prepared, and endodontic treatment was carried out using crown-down technique until size 30, tapered 0.04. Obturation was completed using single cone technique with gutta-percha and AH plus sealer. Cavity access was restored with respective composite resins. Next, teeth were stored in incubator for 24 hours and subdivided into aged and unaged subgroups. Teeth in aged subgroups were subjected to 2500 thermal cycles for 5ºC, 37ºC and 55ºC with 30 seconds dwell time and 5 seconds transfer time. After that, root surfaces of teeth were covered with silicone-based material and placed in boxes filled with acrylic until the cemento-enamel-junction (CEJ) level. They were then tested under Universal Testing Machine until fracture occurred. Samples were then viewed under Leica microscope to determine the fracture pattern and type of fracture. Data analyzed using One-way ANOVA complimented by post hoc Tukey HSD and paired sample T test for fracture strength. Fracture pattern and type of fracture were analyzed using Chi-square test. Level of significance was set at *p*<0.05.

**Results:**

Significant differences were observed (*p*<0.05) with Group 3 demonstrating the highest fracture strength followed by Group 4, Group 1 and lastly Group 2 in both aged and unaged subgroups respectively. A significant decreased in fracture strength was noted in Group 1 and Group 2 (*p*<0.05) as number of thermocycle increased but no significant differences were noted in Group 3 and Group 4 (*p*>0.05). Besides, Group 3 and Group 4 showed higher rate of favorable fracture pattern, followed by Group 1 and lastly Group 2. Most favorable fracture pattern was noted to exhibit horizontal fracture type (86.36%), whereas most unfavorable fracture pattern exhibited vertical fracture type (77.78%).

**Conclusions:**

Endodontically treated teeth restored with new zirconia reinforced rice husk nanohybrid composite (Zr-Hybrid) demonstrated higher fracture strength than commercialized composite resins especially after artificial ageing. Zr-Hybrid showed similar fracture pattern to those of intact teeth with higher rate of horizontal fracture type.

** Key words:**Fracture strength, fracture pattern, composite resin, rice husk, Zirconia.

## Introduction

Root canal treatment is indicated in teeth with pulpal infection which were mainly due to extensive caries and decay in order to preserve the teeth (1). During root canal treatment procedure, a great amount of tooth structure will be lost and consequently weaken the whole coronal tooth structure. Therefore, the teeth require a strong permanent restoration to prevent fracture and to provide a good coronal seal (2,3). Inevitably, endodontically treated anterior teeth need more attention because they are subjected to higher degree of shear forces than posterior teeth due to their anatomical location in the dental arch (4).

For many years, full crown coverage offered the most predictable results for endodontically treated teeth especially incisors (5,6). Undoubtedly, this approach is invasive and will results in removal of large amounts of sound tooth structure. Composite resin has been chosen as one of the direct permanent restorations for endodontically treated teeth due to the fact that it performed better in term of fracture resistance and is able to reinforce weakened tooth structure (3,7). Besides, current adhesive technology also fulfilled the goal of conserving remaining tooth structure and reducing the amount of intervention needed (8).

Owing to the advancement of nanotechnology in adhesive dentistry, nanocomposites have been introduced into the market whereby the size of filler particles incorporated into the resin matrix of composites has been continuously decreased, thus, resulting in nanohybrid and nanofilled materials with improved physical and mechanical properties (9,10). Eco-friendly bio composite has recently gained popularity among researchers in which natural products were incorporated into composite resins. The current study focuses on a new rice husk nanohybrid composite resin reinforced with zirconia nano-powder. Nanohybrid composite resin using rice husk silica as its filler content has shown to possess several advantages such as, being environmental friendly, cost-effective, and of comparable strength (11). The addition of zirconia into composite resin was found to increase the physical strength and fracture toughness of the material (12). Hence, it can be anticipated that this new nanohybrid composite is able to demonstrate better physical properties as compared to commercially available composite resin, thus, boost up the fracture resistance of endodontically treated teeth.

To date, a material that can restore endodontically treated anterior teeth to their original state with respect to their mechanical properties has not yet been found. Most of the published articles focused on the fracture resistance of posterior and maxillary central incisors with different permanent filings, posts and cores, and control groups (2,3,13). Although studies have shown that maxillary central incisors are more prone to fracture (14,15), lateral incisors should also be taken cognizance of. There is still lack of published evidence in the literatures with regard to the fracture strength of endodontically treated lateral incisors restored with different types of composite resin.

Therefore, the aim of this study was to evaluate and compare the fracture strength, fracture pattern and type of fracture of endodontically treated maxillary lateral incisors restored with a new zirconia reinforced rice husk nanohybrid composite and commercially available nanofilled and microhybrid composite resins. The first null hypothesis was that no significant difference would be found in term of fracture strength of endodontically treated maxillary lateral incisors restored with different types of composite resin. Second, there was no significant difference among the fracture pattern of endodontically treated maxillary incisors restored with different types of composite resin. Third, there was no significant difference noted among the types of fracture involved.

## Material and Methods

-Samples Preparation

The present *in vitro* experimental study involved the use of recently extracted maxillary lateral incisors from dental clinics of School of Dental Science, Universiti Sains Malaysia (USM). Ethical approval was obtained from the Human Research Ethics Committee USM (Ref. USM/JEPeM/19120933). PS software version 3.0 was used to calculate the sample size with a standard deviation of 69.1 N from previous study (16). Probability of 0.8 power and alpha of 0.05 were set. 18 samples per group were needed in order to get a difference in fracture strength of 60 N. Therefore, with the addition of 10% drop-out, the estimated number of samples per group was 20 samples and the total estimated sample size was 80 samples.

Eighty mature human permanent maxillary lateral incisors recently extracted from patients within the age group of 30-60 years were collected and inspected under microscope (Leica Micro-system Imaging Solutions, Cambridge, UK) to ensure that there were free from caries, restorations, fracture, or abrasion. The tooth length was measured using a metal ruler (CLR6, Hu-Friedy Mfg. Co. Inc., Chicago, USA) to include teeth with total length of 22 mm (±1 mm) and root length of 13 mm (±1 mm). Radiographic examination (Planmeca, Helsinki, Fineland) was carried out in a buccal and proximal direction to confirm the presence of single canal and mature apical foramen with Type 1 Vertucci’s Classification in all teeth (17). Soft tissue debris and calculus were removed using an ultrasonic scaler (Dentsply Sinora, Bensheim, Germany) and the teeth were immersed in 2.5% sodium hypochlorite solution (NaOCl, Malay-Sino Chemical Industries Sdn. Bhd., Malaysia) for 24 hours at room temperature to remove remaining debris. After that, the teeth were randomly divided into four groups consisting of 20 teeth each:

Group 1: Root canal treatment restored with nanofilled composite resin (Filtek Z350 XT, 3M ESPE, Seefeld, Germany)

Group 2: Root canal treatment restored with microhybrid resin composite (Zmack comp, Zhermack, Badia Polesine, Italy)

Group 3: Root canal treatment restored with zirconia reinforced rice husk nanohybrid composite (Zr-Hybrid, Universiti Sains Malaysia, USM, Malaysia)

Group 4: Intact teeth which acted as control group.

**Figure 1:**
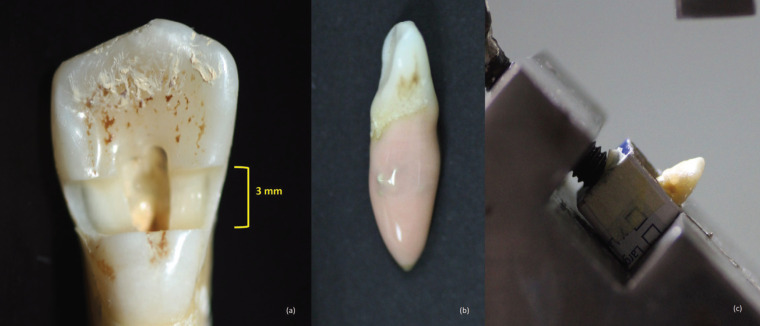
(a). Standardized mesio-palatal-distal (MPD) cavity was prepared on tooth sample. (b). Poly-vinyl siloxane injected over the root surfaces of the tooth sample before reinserted back into the resin block. (c). Tooth was placed on a custom-made metal bar and positioned at 35º angulation in relation to the imaginary vertical axis.

The composition and manufacturer details of each type of composite resin used were listed in Table 1. A standardized mesio-palatal-distal (MPD) cavity was prepared (Fig. 1a) using a straight fissure diamond bur (SF11, Dia-bur, MANI, INC., Japan) with high-speed handpiece for all teeth except for the control group. The cavity was measured using a digital caliper (19975, Shinwa Rules Co., Ltd., Japan) with accuracy of 0.01 mm to ensure that the width of the cavity was 3 mm starting from the cingulum pit towards the coronal and cavity depth of 2 mm. Endodontic treatment was then carried out for Group 1, Group 2 and Group 3 using crown-down technique. First, the cavity was accessed by palatal approach at the cingulum pit using a diamond Endo-Access bur, size 4 (Dentsply Maillefer, USA) and a non-end cutting bur (#851, Dental Burs Australia Pty. Ltd., Australia) with high-speed handpiece to smoothen the walls of the cavity. Canal patency was checked using size 15 K-files (FlexOFiles; Dentsply Maillefer, Switzerland), and root canals were instrumented with NiTi rotary files (ProTaper Next, Dentsply Sirona, Dentsply International Inc., US) up to size X3, 0.04 taper and 1 mm short from the root length. Canals were irrigated copiously using 2.5% sodium hypochlorite (NaOCl) solution and rinsed with 5 ml of 17% ethylenediaminetetraacetic acid (EDTA) solution (Promega Corporation, Wisconsin, USA) to remove smear layer followed by another 5 ml of normal saline solution (RMBIO, Missoula, Montana) as final irrigation to wash out remnants of EDTA. The canals were dried with paper points size 30 (Dentsply, Maillefer, USA) and obturation was done with matched-pair gutta-percha (ProTaper® Next Gutta-Percha Points X3, Dentsply Sirona, Dentsply International Inc., US) and AH plus sealer (Dentsply Maillefer, USA) using single cone technique. The filled gutta-percha was restricted to 1 mm below the cemento-enamel-junction (CEJ).

**Table 1:**
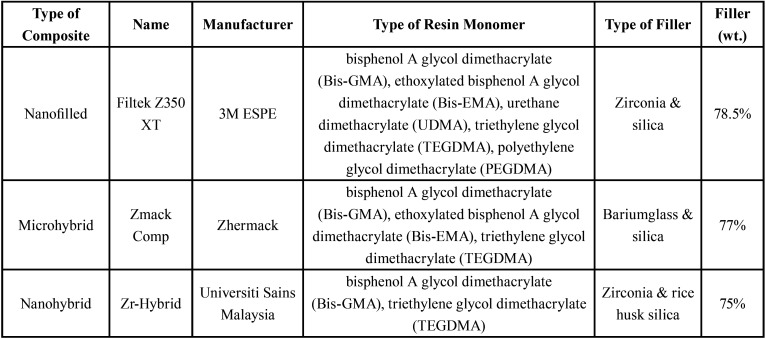
Compositions and Manufacturers of different types of composite resin used in present study.

Following endodontic treatment, the coronal MPD cavity was then acid etched with 35% phosphoric acid (Swiss Tec, Coltene, Malaysia) for 15 seconds, followed by placement of single bond adhesive agent (3M ESPE, 3M Deutschland, Germany) and light cured for 20 seconds using light-emitting diode (LED) light-curing unit Elipar Free Light 2 (3M ESPE, St. Paul, MN, USA) with light intensity of 600 mW/cm2. The teeth were restored using the respective type of composite resin with incremental technique and light cured for 20 seconds as recommended by the manufacturers. Finally, all restorations were polished using composite polishing kit (PN 0310BB, Composite Polishing Kit CA, Shofu, CA, USA).

The teeth were then stored in incubator (ICS200, Yamato Scientific Co., Ltd., Japan) at 37ºC, 100% humidity for 24 hours. For each group, the teeth were subdivided into aged and unaged subgroups. Artificial ageing process involved the aged-group teeth subjected to 2500 thermal cycles using a thermocycling machine (TS Series Liquid, Weiss Technik, North America) in sequential water baths of 5ºC, 37ºC and 55ºC. The dwell time was set at 30 seconds, whereas the transfer time was set at 5 seconds.

-Fracture strength testing

The root portion of tooth samples were wrapped with three layers of aluminum foil (Diamond, LMA-ALU-FOIL, Kumpulan Saintifik F.E. Sdn Bhd., Malaysia) until the CEJ level. Small boxes sized 2 cm × 2 cm × 2 cm were prepared and filled with self-cure acrylic resin (Quick resin, Shofu, Japan). Subsequently, all teeth were immerged vertically along their long axis in the boxes filled with acrylic resin until the level of CEJ and were then removed along the long axis before the acrylic resin is completely set. The aluminum foils were removed and silicone-based impression material, poly-vinyl siloxane (3M™ Imprint™, light body VPS impression, 3M ESPE, 3M Deutschland, Germany), was injected over the root surfaces of the tooth samples (Fig. 1b) which were immediately reinserted back into the resin blocks until the level of CEJ and compressed slightly to allow the material to fill in the space created earlier by the aluminum foil. Excess material that overflowed was removed and thus, a very thin silicone layer that stimulate periodontal ligament (PDL) was formed. The entire procedure was carried out within 4 minutes in order to avoid the material from setting completely and causing gaps at the resin-silicone interface. This allowed a homogenous and standardized silicone layer surrounding the root of each tooth sample. After complete setting of the acrylic resin, the models were taken out from the small boxes and the teeth were placed on a custom-made metal bar (Fig. 1c) which allowed the specimens to be positioned at 35º angulation in relation to the imaginary vertical axis of the teeth. Then, the mounted teeth were subjected to increasing compressive force with a spherical steel tip of 5 mm diameter and downward speed of 1 mm/min, using a Universal Testing Machine (AGS-X, Shimadzu, Japan), until they were fractured, similar to previous study (3). The tip was applied on the center of the restoration which was approximately on the cingulum pit of the teeth. The forces (N) needed to cause fracture of the teeth were recorded and analyzed.

**Figure 2:**
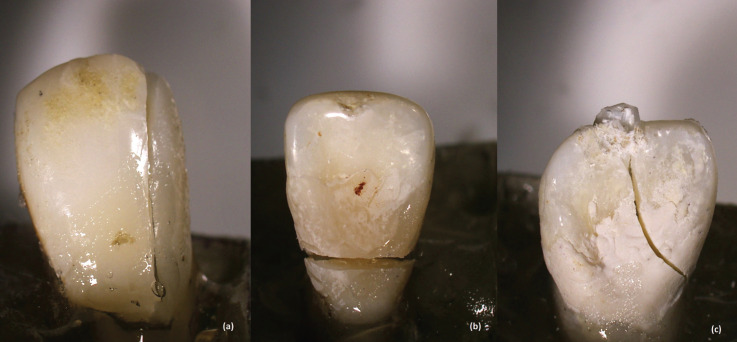
(a). Fracture pattern exhibiting vertical fracture type. (b). Fracture pattern exhibiting horizontal fracture type. (c). Fracture pattern exhibiting oblique fracture type.

The samples were then viewed under microscope (Leica Micro-system Imaging Solutions, Cambridge, UK) at 20x magnification to examine the fracture lines and categorized them into favorable and unfavorable fracture patterns. Fracture lines extending below the CEJ were considered as unfavorable fracture pattern, whereas those extending above the CEJ were considered as favorable fracture pattern. Besides, the types of fracture were also examined to classify them into vertical (Fig. 2a), horizontal (Fig. 2b) or oblique (Fig. 2c) fracture.

-Statistical analysis

Statistical analysis was performed using SPSS version 24.0. Fracture strength was analyzed using One-way ANOVA complemented by multiple comparison between composite resin groups using post hoc Tukey HSD test. Paired sample T test was performed to compare the fracture strength between aged and unaged samples. For fracture pattern and type of fracture line, statistical analyses were done by nonparametric Pearson’s Chi-square test.

## Results

**Figure 3:**
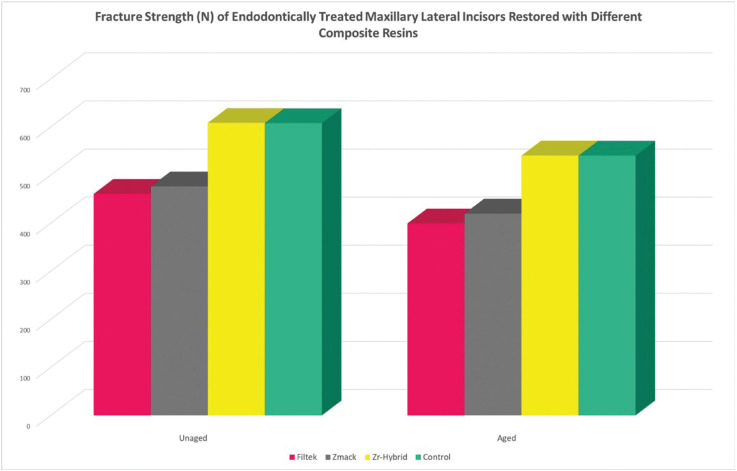
Bar chart of fracture strength of endodontically treated maxillary lateral incisors restored with different composite resins.

The first null hypothesis was rejected. The results of fracture strength of endodontically treated maxillary lateral incisors restored with different composite resins were summarized as mean and standard deviation (SD) in Table 2 and graphically shown in Figure 3. Significant differences were noted (*p*<0.05) in which Group 3 demonstrated the highest fracture strength, followed by Group 4, Group 2 and lastly Group 1 for both aged and unaged subgroups. Post hoc multiple comparison test revealed significant differences among all groups (*p*<0.05) except those between Group 1 and Group 2 (*p*=0.766) and between Group 3 and Group 4 (*p*=0.919) in unaged subgroups; between Group 1 and Group 2 (*p*=0.501) and between Group 3 and Group 4 (*p*=0.998) in aged subgroups. Besides, paired sample T test in Table 3 showed that there were significant differences noted in both Group 1 (*p*=0.001) and Group 2 (*p*=0.003) with and without artificial ageing respectively. However, no significant difference was noted within Group 3 (*p*=0.070) and Group 4 (*p*=0.095).

**Table 2:**
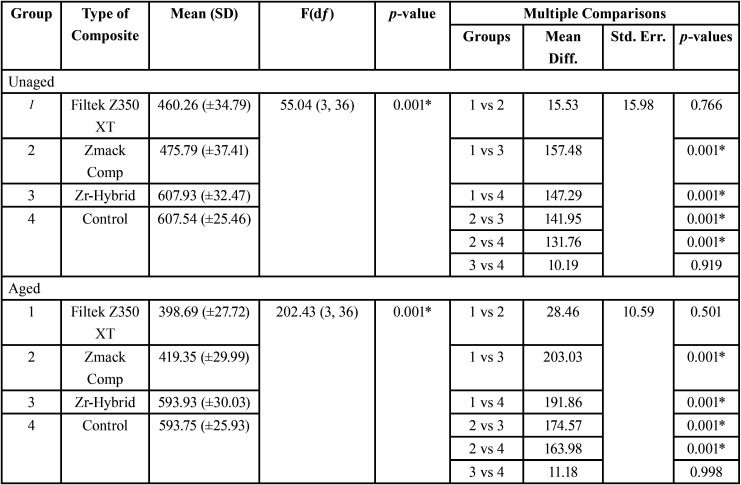
Fracture strength (N) of endodontically treated maxillary lateral incisors restored with different composite resins using One-way ANOVA and multiple comparisons by post hoc Tukey HSD test.

**Table 3:**
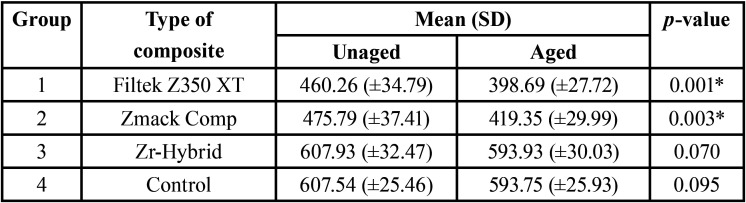
Comparison of fracture strength between unaged and aged endodontically treated maxillary lateral incisors restored with different composite resins using paired sample T test.

The second and third null hypotheses were accepted. In Table 4, Group 1 experienced equal number of favorable (50%) and unfavorable (50%) fracture patterns, among which most of the fractures noted were vertical (50%), followed by horizontal (40%) and lastly oblique (10%). Group 2 experienced higher rate of unfavorable fracture pattern (60%) than favorable (40%). Fractures were found to be vertical (50%), horizontal (40%) and oblique (20%). As for Group 3, higher rate of favorable fracture pattern was noted (70%) among which horizontal fracture was the most common (70%), followed by vertical (30%), and oblique (nil). Lastly in Group 4, most teeth experienced favorable fracture pattern (70%), whereby majority were horizontal (60%), followed by vertical (30%) and oblique (10%). The values of the chi square test were 2.020 and 9.985; whereas *p*-values were 0.587 and 0.125 for fracture pattern and type of fracture respectively. This suggested that the type of composite has no significant bearing on either the fracture pattern or type of fracture. However, it was noted that the most favorable fracture pattern was horizontal, whereas most unfavorable fracture pattern was vertical. There was also a significant association (*p*=0.002) between fracture pattern and the type of fracture based on Table 4.

**Table 4:**
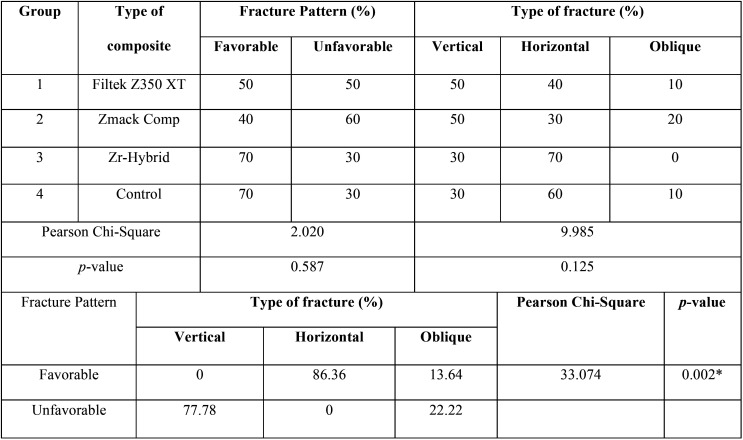
Fracture pattern (%) and type of fracture (%) of endodontically treated maxillary lateral incisors restored with different composite resins using Pearson’s Chi-square test.

## Discussion

Fracture strength or fracture toughness is defined as the resistance of a material to resist crack propagation under high loading force (12). Based on the results of present study, endodontically treated teeth restored with Zr-Hybrid (Group 3) showed comparable fracture strength with intact sound teeth and significantly higher fracture strength than those restored with Filtek (Group 1) and Zmack (Group 2). This can be due to the differences in filler content of composite resins in which higher filler loading was found to increase the mechanical and physical properties (18,19). However, present study demonstrated an interesting result as Zr-Hybrid with the lowest filler content (75% w/w) as compared to Filtek (78.5% w/w) and Zmack (77% w/w) exhibited the highest fracture strength.

One of the possible reasons may be due to the reinforcement of zirconia nano-powder. Zirconia, a ceramic biomaterial, is used widely nowadays in dentistry due to its extremely high strength (20). Utilization of zirconia to enhance the mechanical properties of composite restorations has been reported in the literatures (12,21). In addition, the size of filler particles is another factor that should be considerate. Zmack, a microhybrid composite resin, showed the lowest fracture strength possibly due to its larger particle size compared to Filtek and Zr-Hybrid. Both Filtek and Zr-Hybrid nanoparticles used nano-fillers which has been shown to greatly improved the mechanical properties of composite (22,23). The smaller size of filler particle can be dispersed in higher concentrations, and produces molecules which are more compatible when coupled with resin polymer during polymerization process (23). Thus, this eventually exhibiting excellent physical and mechanical properties.

In present study, teeth were stored in an incubator at 37ºC, 100% humidity for 24 hours prior to thermocycling process. This was due to the fact that curing of composite resins takes up to 24 hours storage in water bath of 37ºC in which thermal stress was found to be rare during this period, while immediate thermocycling would significantly impeded the composite resins from achieving their maximum strength, thus, affecting the reliability of the results (24). Generally, artificial aging of composite resins under thermocycling will accelerates the degradation process and causes a significant decrease in mechanical properties, thus, limiting their long-term success (25). The advantage of using thermocycling is its ability to reproduce a cyclic loading pattern and simulate results of time-consuming clinical trials (26). When comparing the ageing behavior of endodontically treated teeth restored with different composite resins in present study, it was noted that all groups experienced a decrease in fracture strength after artificial ageing. However, an unexpected result was that there was no significant difference in term of fracture strength between aged and unaged Zr-Hybrid. This was probably due to the excellent thermal stability and wear resistance of the fillers, namely, rice husk and zirconia nano-powder in Zr-Hybird composite resin (27-29). This suggested the ability of this new material to maintain excellent physical properties similar to those of natural teeth.

According to the results of the present study, endodontically treated teeth restored with Zmack showed higher rate of unfavorable fracture pattern as compared to other groups. On the other hand, Zr-Hybrid and control intact teeth had similar fracture patterns and it could be postulated that endodontically treated teeth restored with Zr-Hybrid offered a more homogenous stress distribution at the tooth-restoration interface. An association between fracture pattern and type of fracture was found in present study. Teeth exhibited the most favorable fracture pattern were those fracture occurred horizontally above the CEJ level, whereas the most unfavorable fracture pattern demonstrated vertical coronal-radicular fracture. Most fractures in present study occurred in horizontal and vertical directions and this could possibly due to the orientation of enamel rods. Enamel exhibits anisotropic mechanical properties in which the rods are oriented vertically at the incisal edge and horizontally at the center of the teeth slightly above the CEJ level (30). However, they run in oblique direction between these two areas and the area towards the CEJ. Thus, the authors believe that cracks propagate easily in a straight and parallel direction but less so in oblique direction.

All procedures in this study were performed by the same operator in order to provide quality assurance and avoid experimental bias. Besides, present study attempted to simulate PDL and the surrounding tooth supporting structure by covering the roots with thin layer of polyvinyl siloxane instead of directly embedding the teeth into the acrylic resin blocks as mentioned in previous study (13). The thin layer of polyvinyl siloxane represented PDL, whereas the acrylic resin represented alveolar bone. PDL is made up of collagen fibers within a gel-like matrix that allows some mobility of teeth in the socket and transfers high masticatory load from the tooth to the alveolar bone while dissipating the strain energy, thereby protecting the tooth from fracture (31). The method used in present study ensured that the force applied on the teeth was not rigid and mimicked a more natural condition by allowing an even distribution of stress from the crown to the root of the tooth.

It is understood that there are many differences between *in vitro* and *in vivo* situations. First, the forces generated *in vivo* during mastication vary in magnitude, speed and direction, whereas force applied in present study was set at a constant speed and direction. Second, considering the large variation in the degree of incisor inclination among the general population (32,33), it is difficult to make a definite assumption based on current results since the fracture strength of the teeth might differ according to different incisor relationships. Therefore, it is impossible to directly extrapolate the results obtained in a controlled lab study to clinical situation necessitating. Further *in vivo* and clinical trials are required before Zr-Hybrid could be used as an option to permanently restore endodontically treated teeth.

## Conclusions

Within the limitation of this study, it can be speculated that endodontically treated teeth restored with new zirconia reinforced rice husk nanohybrid composite showed higher fracture strength than commercially available nanofilled and microhybrid composite resins with similar fracture pattern to those of intact teeth especially after artificial ageing. Besides, most favorable fracture pattern was found to exhibit a horizontal fracture type.
